# Titanium (IV) oxide anatase nanoparticles as vectors for diclofenac: assessing the antioxidative responses to single and combined exposures in the aquatic macrophyte *Egeria densa*

**DOI:** 10.1007/s10646-023-02646-7

**Published:** 2023-03-31

**Authors:** Maranda Esterhuizen, Mariia Lutsko, Youngsam Kim, Hakwon Yoon, Chang-Beom Park, Young Jun Kim, Stephan Pflugmacher

**Affiliations:** 1 Ecosystems and Environment Research Programme, Faculty of Biological and Environmental Sciences, Niemenkatu 73, University of Helsinki, 15140 Lahti, Finland; 2 Helsinki Institute of Sustainability Science (HELSUS), Fabianinkatu 33, 00014 Helsinki, Finland; 3 Clayton H. Riddell Faculty of Environment, Earth, and Resources, University of Manitoba, Wallace Building, 125 Dysart Road, Winnipeg, MB R3T 2N2 Canada; 4 Korea Institute of Science and Technology Europe (KIST Europe) Forschungsgesellschaft GmbH, Joint Laboratory of Applied Ecotoxicology, Environmental Safety Group, Universität des Saarlandes Campus E7 1, 66123 Saarbrücken, Germany; 5 Department of Biotechnology, Technische Universität Berlin, Gustav-Meyer-Allee 25, 13355 Berlin, Germany; 6 Environmental Exposure & Toxicology Research Center, Korea Institute of Toxicology, 17, Jegok-gil, Jinju, 52834 Republic of Korea

**Keywords:** Oxidative stress, Biotransformation, Nanomaterials, Pharmaceutical pollution, Primary producer, Macrophyte

## Abstract

Titanium dioxide, frequently used in commonplace products, is now regularly detected in aquatic environments. Understanding its toxic effects on native biota is essential. However, combined toxicity with commonly occurring pollutants, such as the pharmaceutical diclofenac, may provide more insight into environmental situations. Therefore, the present study aimed to evaluate the effects of titanium dioxide and diclofenac, individually and combined, on the macrophyte *Egeria densa*. Diclofenac uptake and removal by the macrophyte were assessed. Diclofenac and titanium dioxide were mixed prior to exposure to allow binding, which was assessed. Toxicity of the individual compounds and the combination was evaluated by assaying enzymes as bioindicators of biotransformation and the antioxidative system. Cytosolic glutathione S-transferase and glutathione reductase activities were increased by diclofenac, titanium dioxide, and the combination. Both enzymes’ activities were more significantly elevated by diclofenac and the combination than nanoparticles alone. Microsomal glutathione S-transferase was unaffected by diclofenac exposure but inhibited with titanium dioxide and the mixture. Diclofenac elicited the most significant response. Based on the data, the cytosolic enzymes effectively prevented damage.

## Highlights

- mGST was inhibited by TiO_2_, but DCF exposure was insignificant.
- cGST and GR activities increased with DCF exposure.
- No synergistic effect on cGST and GR with combined exposure.
- DCF, rather than TiO_2_, was responsible for oxidative stress-related toxicity.
- Macrophytes remained healthy despite exposures at high concentrations.

## Introduction

Various engin eered nanomaterials are produced in hundreds of tons per year (Hendren et al. 2011). The nanotechnology industry has enormous growth prospects and opportunities for commercial development due to the vast range of applications of nanomaterials and particles. Nanoparticles (NPs), which are in the 10^−9^ m range, are used in electronics, medical and pharmaceutical industries, consumer goods, food production, as well as military applications (Khan et al. 2019). NPs are of great importance for scientific studies as a middle link between bulk materials and atomic structures. These minute particles have a much larger surface-to-volume ratio than similar masses of larger-scale materials. As the surface area per mass of material increases, considerably more material can come into contact with surrounding substances. The larger the surface area, the greater the substance’s reactivity, allowing improved catalysts to be created (Lien et al. 2015); e.g., the drastic property changes of gold NPs as oxidants compared to gold macroparticles. NPs’ mechanical and magnetic properties also differ from their regular-shaped counterparts, meaning that adhesion and capillary forces exceed macroscopic forces, including superparamagnetic forces (Wahajuddin and Arora 2012). Thermal and optical properties also shift; i.e., with decreasing size, surface energy increases, thus reducing melting points (Mashayekh and Dorranian 2014).

The use of titanium (IV) oxide (or titanium dioxide, TiO_2_-NPs) is increasing due to its nanosized features, low toxicity, biocompatibility, intrinsic properties, and manufacturing techniques (Jarosz et al. 2016; Kafshgari et al. 2019; Molaeirad et al. 2015; Naseri et al. 2015). These nanomaterials are also recognized for their high refractive index, light scattering capabilities, and photocatalytic activities in the presence of UV with equal or higher energy than its bandgap energy. TiO_2_-NPs occur in three crystalline phases, brookite, rutile, and anatase, with the latter showing a more extensive band gap and, thus, the highest photocatalytic effects (Skocaj et al. 2011). Therefore, TiO_2_-NPs are one of the most commonly used metal oxides (Jovanović 2015). They are widely used in paints, floor coatings, paper cosmetics, cleaning products, and sunscreens. However, some studies have shown contradictory evidence regarding the toxicity and long-term stability of these NPs, as reviewed by Skocaj et al. (2011). Among others, TiO_2_ has been implicated in oxidative stress induction as well as cellular dysfunction as it produces hydroxyl radicals with cytotoxic effects. However, the response of the antioxidative system in this regard remains unexplored.

Engineered NPs, including titanium-based nanomaterials, have been detected in the environment at concentrations up to 48 ng/ml (Tovar-Sánchez et al. 2013); however, Environmental Fate Modeling predicts this level to be closer to 10000 ng/ml (Maurer-Jones et al. 2013). These particles may enter the environment in various ways, primarily through industrial wastewater. NP-containing personal care products may also deposit in domestic wastewater and, from there, enter sewage sludge (Coll et al. 2016; Sun et al. 2016). Once in aquatic ecosystems, NPs could mix with other pollutants, including pharmaceuticals, and could affect keystone species in various ways. According to Thiagarajan et al. (2021), who reviewed the interactions between nanomaterial, pharmaceuticals, and nano/microplastics, these compounds are commonly detected in aquatic environments globally and bound to co-occur and interact. As the adverse effects of pharmaceuticals have already been recognized (Fent 2008; Mezzelani and Regoli 2022), it becomes vital to understand the impact of NPs on biota and in combinations with pharmaceuticals detected in surface waters globally. One such globally detected drug is diclofenac (DCF) (Li 2014). The environmental concentration of DCF in aquatic environments varies considerably (Lonappan et al. 2016). Fekadu et al. (2019) reported mean diclofenac concentrations detected in European waters to range from approx. 3 to 5 ng/ml and in African waters from approx. 5 to 7 ng/ml.

As a first step in evaluating the toxicity of NPs as well as their combined toxicity as vectors for pharmaceuticals, TiO_2_-NP in its anatase form was selected for this study due to its wide use. The toxicity of TiO_2_-NPs, DCF, and a combination of the NPs and the pharmaceutical, was evaluated on the ecologically essential macrophyte species *Egeria densa*. Macrophytes serve as primary producers, as well as habitat, shelter, and breeding space for other organisms contributing to the overall biodiversity. They also influence the nutrient cycles in aquatic environments (Bakker et al. 2016; Esteves 1998; Kennedy et al. 2004; Pott and Pott 2003; Thomaz and Cunha 2010); and are excellent bioindicators (Ravera 2001). *E. densa* was selected based on its advantageous features, including rapid growth and natural ubiquity, and due to the limited information on adverse effects on this macrophyte caused by NPs. Additionally, information on how *E. densa* responds to NPs and pharmaceuticals may help evaluate its potential utility in the phytoremediation of water contaminated with these substances. *E. densa* has been shown to efficiently remediate NPs such as Ag-NPs (Bernas et al. 2017) and pharmaceuticals (De Morais Calado et al. 2019). However, information on the phytoremediation of DCF and TiO_2,_ as well as a combination of the two, is lacking.

Toxicity is often mediated by oxidative stress, as an organism’s inability to eliminate increased reactive oxygen species (ROS) at a cellular level would lead to severe adverse effects and eventual mortality (Sarkar et al. 2014). Fluctuations in the antioxidative enzyme responses are often used as bioindicators of oxidative stress (Gutteridge 1995). In the present study, glutathione reductase (GR) and glutathione S-transferase (GST) were selected as biomarkers. GR is an antioxidative defense enzyme involved in recycling glutathione to combat ROS generated from xenobiotics. GST is a crucial enzyme in phase II of the biotransformation system, which is vital in eliminating xenobiotics.

The study, therefore, aimed to evaluate the toxicity of TiO_2_ and DCF as well as combined toxicity in *E. densa* by evaluating GST and GR as biomarkers of antioxidative response to xenobiotic exposure.

## Materials and methods

### Chemicals and reagents

DCF (sodium salt, ≥99%) was bought from Cayman Chemical Company (Michigan, USA). Stock solutions were prepared in pure ethanol as required, and further dilutions were conducted in the cultivation/exposure media of choice.

Anatase TiO_2_ (100% anatase, <25 nm, specific surface area 45- 50 m^2^/g, purity 99.7%) was purchased from Sigma-Aldrich Co. Ltd. (Steinheim, Germany) and was from the same batch as used by Okupnik et al. (2015) who characterized the material in terms of size, morphology, zeta potential, z-average hydrodynamic diameter, and the polydispersity index (PDI).

All chemicals used for exposure and analysis were of analytical-grade quality and were obtained from Sigma-Aldrich Co Ltd. (Steinheim, Germany) unless stated otherwise.

### Egeria densa

*E. densa* (strands of 10–20 cm) was purchased from Extraplant (Extragroup GmbH, Germany) and cultivated in a glass tank (100 cm × 60 cm × 60 cm) at 24 ± 1 °C. The plants were grown under cool white fluorescent light with a light intensity of 38 μE/m^2^/s and a 14:10-h light-dark photoperiod. The culture media consisted of modified Provasoli’s culture medium containing CaCl_2_ (0.2 g/l), NaHCO_3_ (0.106 g/l), and sea salt (0.1 g/l) in de-ionized water (Vilvert et al. 2017). The macrophytes were acclimated to laboratory conditions for seven days before the exposures. DCF uptake into *E. densa* and removal from the media were evaluated prior to the exposure experiments to establish its suitability for this investigation. Three-centimeter *E. densa* strands were exposed to 250 ng/ml DCF in beaker experiments against controls for 96 h under the same conditions as during acclimation (*n* = 5). Plant and media samples were collected after 24, 48, 72, and 96 h. DCF was extracted from the plant tissue as detailed by De Morais Calado et al. (2019), and DCF was quantified as described in section 2.4.

### Exposure setup

Three treatment solutions were prepared. The first consisted of DCF diluted to 250 ng/ml in the *E. densa* cultivation media. Concentrations previously reported for DCF in wastewater and the environment served as guidance for choosing this exposure concentration (Esterhuizen-Londt et al. 2017). The second exposure solution consisted of 250 ng/ml DCF combined with 1 mg/ml TiO_2_-NP anatase in cultivation media, and the third consisted of 1 mg/ml TiO_2_-NP anatase only in the cultivation media. The control consisted of the macrophyte cultivation media without additions of other chemicals. A sample from each prepared exposure solution was collected for qualitative analysis with liquid chromatography-tandem mass spectroscopy (time 0). The solutions were stirred for 24 h in the dark, and a second sample was taken for analysis to measure any degradation or binding (time 24).

After the 24 h binding/degradation study, the treatment solutions were decanted in 100 ml beakers in replicates of five, and a 20 ± 1 cm strand of *E. densa* was added to each replicate and exposed for 24 h under the same conditions as during cultivation. After 24 h of exposure, another media sample was taken for quantitative analysis (time 48). The plant material was removed from the treatments, washed in distilled water, dried, and snap-frozen in liquid nitrogen. The samples were stored at −80 °C until further processing to evaluate the enzyme activities.

### Quantitative analysis of diclofenac

DCF was quantified on a 1200 infinity series liquid chromatography (Agilent, Waldbronn, Germany) coupled to triple quadrupole mass spectrometry (model 6460, Agilent) (LC-MSMS) with electron spray ionization (Jet Stream, Agilent) using a Kinetex™ C18 reverse phase column (2.1 × 100 mm, 1.7 U, 100 Å, Phenomenex, Aschaffenburg, Germany). The LC-MSMS settings and protocol were detailed by Esterhuizen-Londt et al. (2017) with a 0.5 pg on column (S/N > 5) limit of quantification. Prior to analysis, all samples were centrifuged at 10,000 × *g* at 10 °C for 30 min.

### Enzyme extraction and activity assays

The enzymes were extracted according to Pflugmacher (2004). In short, the frozen plant material was pestled to a refined power using liquid nitrogen, and 1.5 g thereof was suspended in 0.1 M potassium phosphate buffer (pH 6.5) containing 20% glycerol, 1.4 mmol/l dithioerythritol, and 1 mmol/l ethylenediaminetetraacetic acid. The samples were stirred for 20 min before centrifugation at 5400 × *g* (4 °C) for 10 min to remove cell debris. The supernatant was centrifuged at 86,900 × *g* (4 °C) for 60 min to collect the microsomal fraction. The supernatant was subjected to ammonium sulfate precipitation (35–80%), collecting the pellet after centrifugation. The cytosolic enzymes, now contained in the pellet, were suspended in a 20 mM pH 7 sodium phosphate buffer. The samples were desalted using Sephadex NAP-10 columns (GE Healthcare, Little Chalfont, UK).

The protein concentrations of the two fractions of each sample were measured according to Bradford (1976). The enzymatic activities of GST (microsomal and cytosolic) and GR (cytosolic) were measured spectrophotometrically (Infinite M200, Tecan, Männedorf, Switzerland) and expressed in the SI units of kat/mg protein. GST activity (EC 2.5.1.18) was assayed by measuring an increase in optical density at 340 nm following to conjugation of glutathione and 1-chloro-2,4-dinitrobenzene (Habig et al. 1974). GR activity (EC 1.6.4.2) was measured as a decrease at 340 nm as nicotinamide adenine dinucleotide phosphate (NADPH) was consumed (Carlberg and Mannervik 1985).

### Statistical analyses

All statistical analyses were performed using IBM® SPSS® Statistics 28.0.0.0 (190) (2021). The DCF concentrations quantified in the treatments were compared with the independent samples *t* test, and the DCF concentrations quantified over time were compared using the paired-samples *t* test. The enzyme activity data did not meet the requirements of sphericity and homogeneity, and thus, the non-parametric Kruskal-Wallis test with pairwise comparisons was used, observing an alpha value of 0.05 after Bonferroni correction (Sokal and Rohlf 1987).

## Results and discussion

### DCF degradation and binding to TiO_2_

**Fig. 1:**
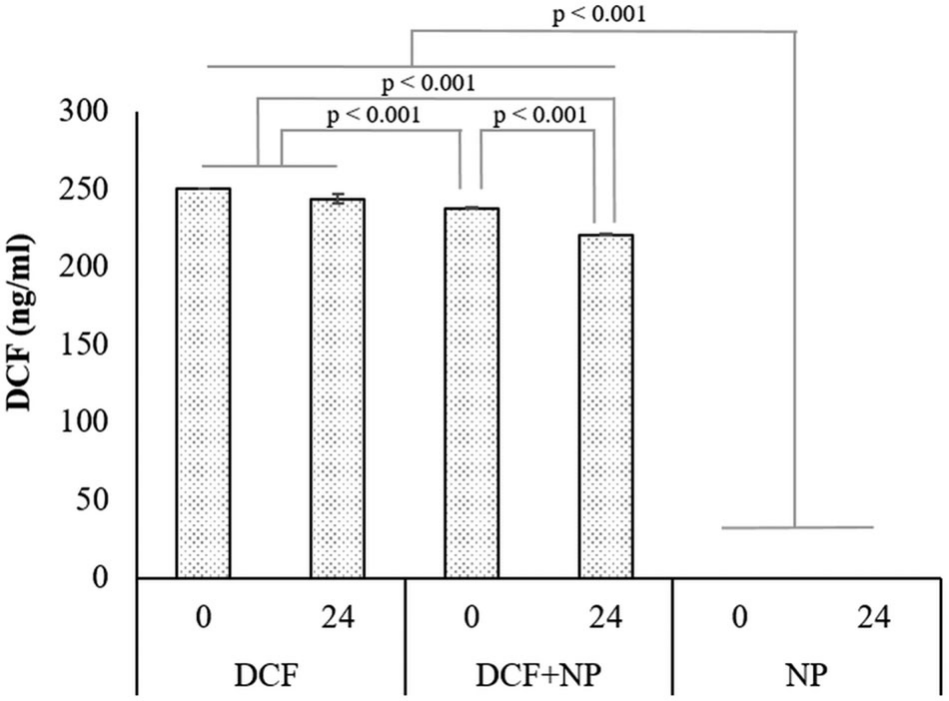
Concentration of free diclofenac. Quantitative analysis of soluble diclofenac (DCF) at the start and end of the 24-h incubation period on its own (DCF, positive control), in the presence of titanium dioxide (DCF + NP), and the nanoparticles on its own (NP, negative control). Bars represent average DCF concentration ± standard deviation as measured by liquid chromatography-tandem mass spectroscopy (*n* = 3)

Under the experimental conditions for the binding study prior to exposure with the macrophyte, the DCF concentration (Fig. 1), without TiO_2_-NPs, remained unchanged after 24 h (*p* = 0.060). However, in the presence of TiO_2_-NPs, the DCF concentration decreased by 11.9% (*p* < 0.001). DCF degradation was not found in the treatments without TiO_2_-NPs. Therefore, the undetected 11.9% in the presence of TiO_2_-NPs was likely due to binding to the NPs. Considering the concentrations of DCF (250 ng/ml in 100 ml = 25 µg) and the TiO_2_-NPs (1 mg/ml in 100 ml = 100 mg) per replicate, 2.98 µg DCF was bound per 100 mg of TiO_2_ (29.8 µg/g) after 24 h. TiO_2_ photocatalysis of DCF was previously demonstrated by Rizzo et al. (2009). However, degradation can be excluded as these experiments were conducted in the dark. Similar to the findings in the present study, Rizzo et al. (2009) reported that after 30 min, 14% of the DCF (5 µg/ml) was adsorbed to the TiO_2_ (0.2 mg/ml) in the dark, and thereafter saturated under the prevailing conditions. No DCF contamination was detected in the pure TiO_2_-NP treatments.

### Enzymatic responses of *Egeria densa*

Studies regarding the adverse effects of DCF on macrophytes are limited. However, the available studies indicate moderate toxicity as the EC_50_ of DCF was determined to range from 7 to 350 µg/ml for microalgae such as *Desmodesmus subspicatus and Pseudokirchneriella subcapitata, as well as* macrophytes such as *Lemna minor, Nasturtium officinale, and Callitriche platycarpa* (Cleuvers 2003; Ferrari et al. 2003; Joachim et al. 2021). In the present study, a DCF exposure concentration of 250 ng/ml was used. DCF was internalized by the macrophyte dose-dependently over time at a rate of 3.2 ng/g/h after the first 24 h, which decreased to 1.5 ng/g/h after 96 h (Fig. S1). Accordingly, at the conclusion of all exposures, all plants in all treatments visually appeared healthy, and no chlorosis or necrosis could be observed. Nevertheless, DCF toxicity is said to increase under the sunlight due to the toxicity of its phototransformation byproducts, mainly 2-[(2-chlorophenyl)amino] benzaldehyde (CPAB) for which an EC_50_ of 4800 ng/ml was determined, which is 10-times lower than that of DCF (48100 ng/ml) for *Scenedesmus vacuolatus* (Schulze et al. 2010). For the generation of phototransformation byproducts, Schmitt-Jansen et al. (2007) indicated that for *S. vacuolatus*, maximal toxicity was achieved after 53 h of light exposure to 50,000 ng/ml of DCF. Furthermore, Andreozzi et al. (2003), studying the photodegradation of DCF, indicated that the half-life was around five days under constant light conditions. Thus, the relatively low DCF concentration (250 ng/ml) and the short incubation under light (14 h) used in the present study would not induce substantial adverse effects by CPAB generation.

**Fig. 2:**
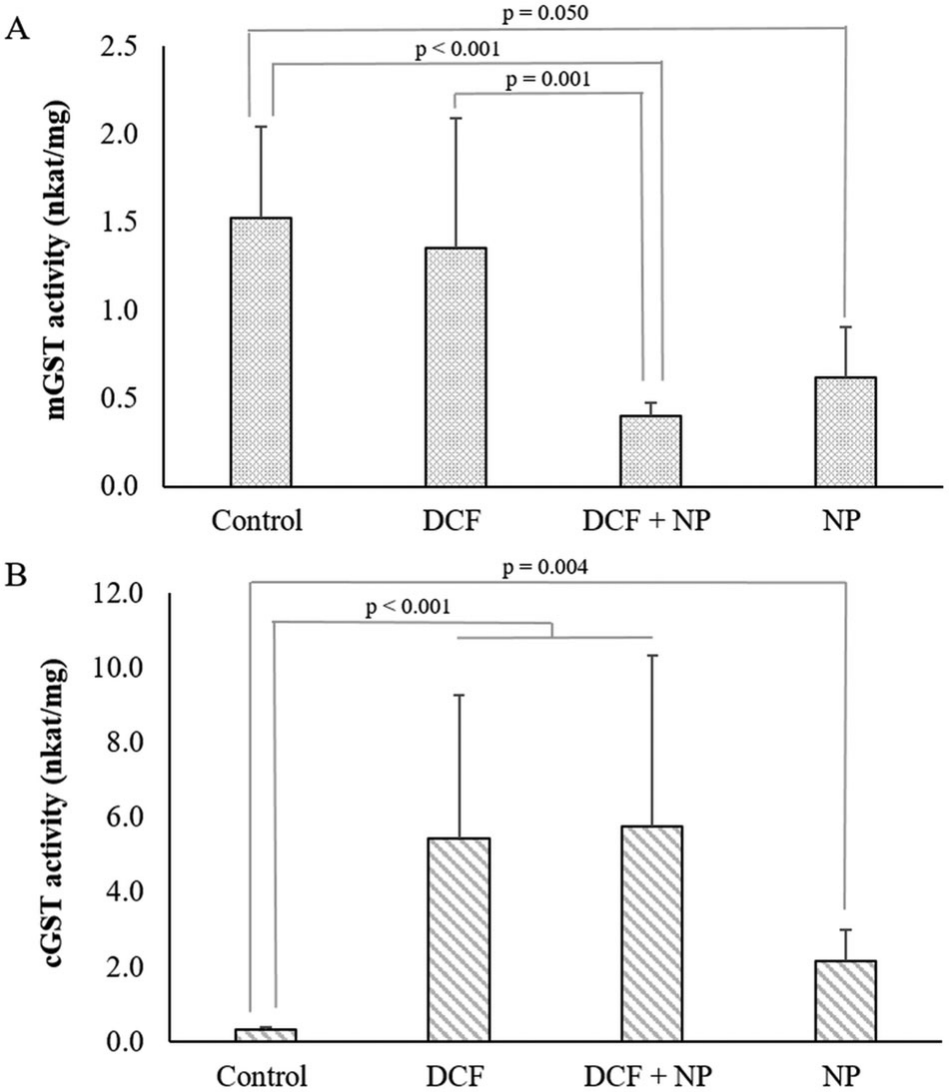
Glutathione S-transferase activities. Activities of the (**A**) microsomal and (**B**) cytosolic glutathione S-transferase (GST) of *Egeria densa* exposed to diclofenac (DCF), TiO_2_-NP pre-exposed to DCF (DCF + NP), and TiO_2_-NP. Bars present average enzyme activity ± standard deviation (*n* = 5)

When exposing *E. densa* to DCF, the microsomal GST (mGST) activity was not elevated (*p* = 1) at the applied concentrations. However, with exposure to the TiO_2_-NP alone or in combination with DCF, the mGST activity was inhibited (Fig. 2A). Compared to the control, the *E. densa* mGST activity was reduced by 73.8% (*p* < 0.001) with exposure to DCF in combination with TiO_2_-NP and 59.8% with NPs (*p* = 0.050). Since most microsomal enzyme substrates are lipophilic compounds (Yu 2002), the detoxification mechanism will be limited for hydrophilic compounds such as DCF. The data indicate that the mitochondrial detoxification of TiO_2_ is limited; however, more so in the presence of DCF. Thus, cGST is more likely to be involved in detoxifying the two compounds.

The activity of cGST (Fig. 2B), in contrast, was significantly elevated with exposure to all three treatments. Exposure to DCF increased the enzyme activity 16.6 ± 0.5-fold with DCF as well as DCF-TiO_2_-NP (*p* < 0.001). NP exposure caused a 6.4-fold increase in cytosolic GST (cGST) activity (*p* = 0.004). In general, the phi and tau classes of plant-specific cGST are predominantly present to detoxify and restrict the effects of xenobiotics (Kumar and Trivedi 2018). In *Solanum lycopersicum* L., GST activity increased 1.5-fold with exposure to 1500 ng/ml DCF (Sousa et al. 2021).

Previously, GST activity in the macrophyte *T. latifolia* associated with DCF exposure was studied by Bartha et al. (2014). Using a similar photometric analytical method, the authors reported no elevation of the GST activity after 24 h of exposure to 1000 ng/ml DCF. However, the activity was significantly induced after 72 h. In contrast to the results presented here, Alkimin et al. (2020) reported inhibition of the GST level in *L. minor* exposed to 375 ng/ml, 750 ng/ml, and 1500 ng/ml DCF. Changes in antioxidant enzyme activity and its related gene expression are associated with antioxidant capacity and response in time courses (Dinler et al. 2014). Interestingly, Varela-Valencia et al. (2014) reported induced expression of the GST gene with anatase TiO_2_ after only 6 h. However, in the present study, only the activities of cGST increased.

GSTs are often associated with antioxidative defense mechanisms and biotransformation, as reviewed by Edwards et al. (2000). However, in the present study, the mGSTs were inhibited with exposure to the NPs and in combination with DCF, which may have led to elevated oxidative stress. Nanomaterials have been shown to bind to some proteins, such as bovine serum albumen (Giacomelli et al. 1997), and specific enzymes, such as lysozyme (Xu et al. 2010) and lactate dehydrogenase (MacCormack et al. 2012), leading to structural changes and inhibition (Xu et al. 2010).

**Fig. 3:**
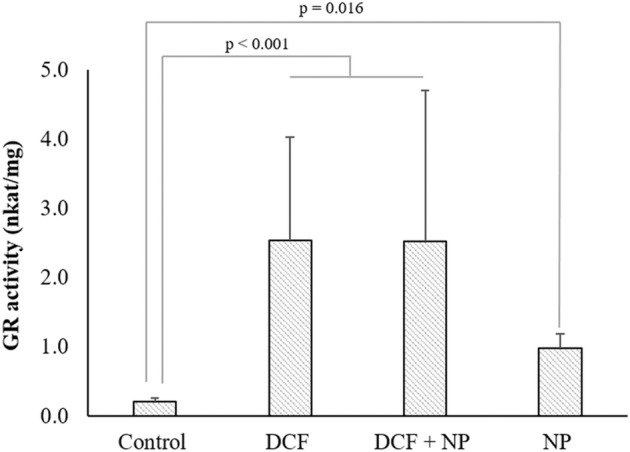
Glutathione reductase activities. Glutathione reductase (GR) activity of *Egeria densa* exposed to diclofenac (DCF), TiO_2_-NP pre-exposed to DCF (DCF + NP), and TiO_2_-NP. Bars present average enzyme activity ± standard deviation (*n* = 5)

Considerating the key role GR plays in the cellular control of oxidative stress by the generation of glutathione (GSH), *E. densa*’s GR activity (Fig. 3) responded in the same manner as cGST (Fig. 2B). The GR activity increased by 12.2-fold with exposure to DCF and a combination of DCF + NP (*p* < 0.001). Exposure to the TiO_2_-NP only resulted in a 4.7-fold increase in activity. Since TiO_2_ nanoparticles are considered one of the safest and low-toxic materials, the significant increase in GR activity indicated that exposure to TiO_2_ induced substantial effects. Okupnik and Pflugmacher (2016) also reported a significant increase in the GR activity in *Hydrilla verticillata* with exposure to anatase TiO_2_. In the study by Bartha et al. (2014), the GR activity increased in shoots but not in roots of *T. latifolia* exposed to 1000 ng/ml DCF for seven days. Sousa et al. (2021) reported increased GR activity in both roots and shoots of *S. lycopersicum* L. However, the exposure concentration was up to 5000 ng/ml DCF for five weeks.

In contrast to mGST, cGST and GR activities were not inhibited with exposure to the NPs alone. Studies have proposed preferential binding of specific NPs to certain enzymes (Bayraktar et al. 2006; Fischer et al. 2002). Our data supports this as mGST and cGST are structurally distinct isozymes and evolutionarily diverse (Vaish et al. 2020). Some studies have also discussed the possibility that high ROS concentrations induce DNA and RNA damage, lipid peroxidation, and protein oxidation/denaturation with consequent enzyme inhibition (Alkimin et al. 2020). Furthermore, TiO_2_ particles are known to interact with phospholipids through possible binding by hydroxyl groups of the terminal glycerol (Le et al. 2014). Another study showed that TiO_2_ made pits in membranes (Batiuskaite et al. 2022). These studies demonstrate that TiO_2_ affects the integrity of membranes, which is necessary for mGST to remain functional, potentially explaining the loss of activity observed here. Investigating the role of lipid peroxidation related to the functionality of the mGSTs in the future is essential in understanding potentially associated oxidative stress. Nevertheless, the preferential binding of NPs seems more plausible and warrants future research.

To summarize the results, the activity of mGST was inhibited by the TiO_2_ nanoparticles, and the effect of DCF was insignificant. Oppositely, the activities of cGST and GR were increased by DCF, but no synergistic effect was found with the TiO_2_ nanoparticles. However, it is known that cytosolic GSTs are more involved in detoxification than mitochondrial and microsomal GSTs (Dasari et al. 2018). Therefore, it is estimated that the major contributor to the elevated antioxidant response observed in aquatic macrophyte *E. densa* is DCF rather than the TiO_2_ nanoparticles. In general, the macrophyte was able to cope with the adverse effects associated with exposure to these high concentrations of DCF and TiO_2_, as well as a combination of the two, as visually evident from lack of chlorosis or necrosis and the plants continued to grow throughout the exposure period.

The concentration of pollutants utilized in the present study exceeds that of currently measured concentrations; however, these concentrations of titanium dioxide and DCF may increase in the future to these values. This study provides a brief insight into the toxic effects of titanium dioxide, diclofenac, and their combined toxicity on the antioxidant and the biotransformation system in the model aquatic macrophyte *E. densa*. Assessing other physiological markers, such as, for example, total reactive oxygen species, chlorophyll content, gene expression of the biotransformation and antioxidative enzymes, would provide more information on the toxicity of the individual xenobiotics and combined toxicity. Furthermore, additional information on combined toxicity is needed; therefore, ecotoxicological investigations with various combinations and mixtures are required.

## Conclusion

The study shows that even at concentrations higher than environmentally detected, the macrophyte *E. densa* responds to environmental pollutants such as nanoparticles and pharmaceuticals, in this case, TiO_2_ and DCF, adequately by elevating antioxidative responses and biotransformation processes to avoid adverse effects. Further studies are required to understand why mGST but not cGST is inhibited by the nanomaterials. The study illustrates the discrepant results when comparing the physiological outcomes with exposure to the compounds in single and mixtures. This information becomes essential when considering the cocktails of pollutant mixtures in the environment and thus emphasizes the importance of considering the synergistic and antagonistic effects of mixture effects in future experiments.

## Supplementary information

Supplementary Fig

Supplementary Information
