# Supplementary figures and images for: Titanium (IV) oxide anatase nanoparticles as vectors for diclofenac: assessing the antioxidative responses to single and combined exposures in the aquatic macrophyte Egeria densa
Source: Ecotoxicology. 2023 Mar 31;32(3):394–402. doi: 10.1007/s10646-023-02646-7 (PMC10102128; doi:10.1007/s10646-023-02646-7)

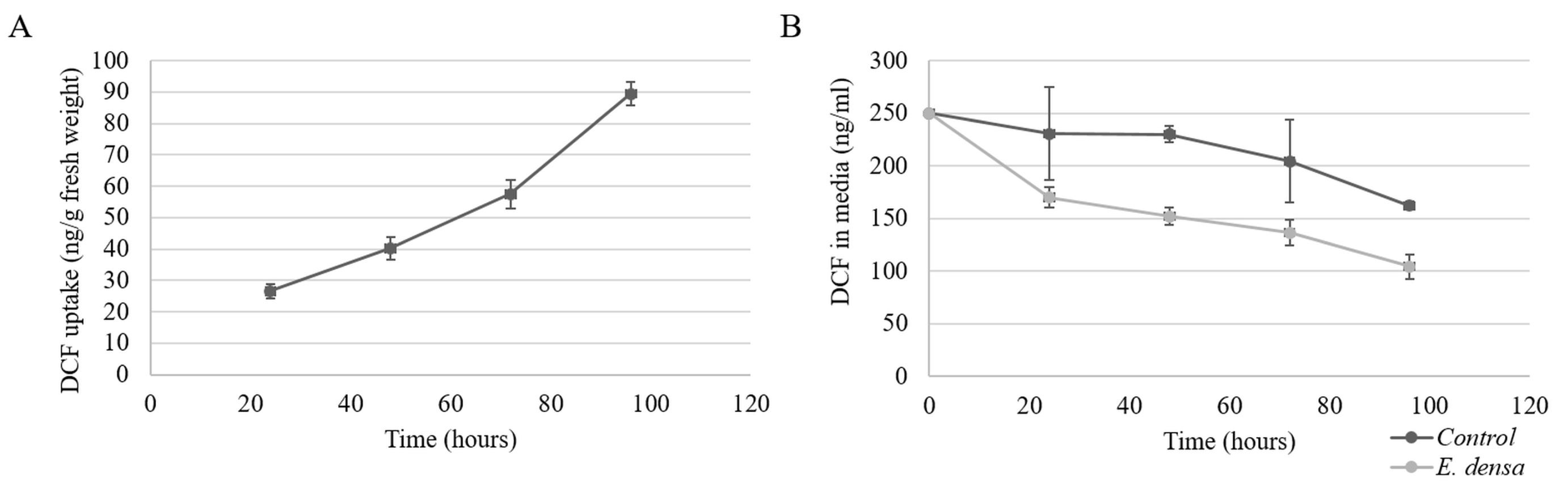

Supplement: Supplementary file 1 — Supplementary Fig [file 10646_2023_2646_MOESM1_ESM.tif]
