# Supplementary material for: Titanium (IV) oxide anatase nanoparticles as vectors for diclofenac: assessing the antioxidative responses to single and combined exposures in the aquatic macrophyte Egeria densa
Source: Ecotoxicology. 2023 Mar 31;32(3):394–402. doi: 10.1007/s10646-023-02646-7 (PMC10102128; doi:10.1007/s10646-023-02646-7)
Supplement: Supplementary file 2 — Supplementary Information [file 10646_2023_2646_MOESM2_ESM.docx]

**Figure S1:** A) DCF uptake by the aquatic macrophyte *Egeria densa* as ng DCF per gram of fresh-weight plant material and B) the natural degradation of DCF (control) and removal of DCF from the media in the presence of *E. densa*. Data present average DCF concentration ± standard deviation (n = 5).
